# Semiconductor–metal transition in Bi_2_Se_3_ caused by impurity doping

**DOI:** 10.1038/s41598-023-27701-5

**Published:** 2023-01-11

**Authors:** Takaki Uchiyama, Hidenori Goto, Eri Uesugi, Akihisa Takai, Lei Zhi, Akari Miura, Shino Hamao, Ritsuko Eguchi, Hiromi Ota, Kunihisa Sugimoto, Akihiko Fujiwara, Fumihiko Matsui, Koji Kimura, Kouichi Hayashi, Teppei Ueno, Kaya Kobayashi, Jun Akimitsu, Yoshihiro Kubozono

**Affiliations:** 1 Research Institute for Interdisciplinary Science, Okayama University, Okayama, 700-8530 Japan; 2 Advanced Science Research Center, Okayama University, Okayama, 700-8530 Japan; 3 Faculty of Science and Engineering, Kindai University, Osaka, 577-8502 Japan; 4 Department of Nanotechnology for Sustainable Energy, Kwansei Gakuin University, Sanda, 669-1330 Japan; 5 Institute for Molecular Science, UVSOR Synchrotron Facility, Okazaki, 444-8585 Japan; 6 Department of Physical Science and Engineering, Nagoya Institute of Technology, Nagoya, 466-8555 Japan; 7 Japan Synchrotron Radiation Research Institute (JASRI), SPring-8, 1-1-1 Kouto, Sayo, Hyogo 679-5198 Japan

**Keywords:** Topological insulators, Electronic and spintronic devices, Electronic properties and materials, Two-dimensional materials

## Abstract

Doping a typical topological insulator, Bi_2_Se_3_, with Ag impurity causes a semiconductor–metal (S-M) transition at 35 K. To deepen the understanding of this phenomenon, structural and transport properties of Ag-doped Bi_2_Se_3_ were studied. Single-crystal X-ray diffraction (SC-XRD) showed no structural transitions but slight shrinkage of the lattice, indicating no structural origin of the transition. To better understand electronic properties of Ag-doped Bi_2_Se_3_, extended analyses of Hall effect and electric-field effect were carried out. Hall effect measurements revealed that the reduction of resistance was accompanied by increases in not only carrier density but carrier mobility. The field-effect mobility is different for positive and negative gate voltages, indicating that the *E*_F_ is located at around the bottom of the bulk conduction band (BCB) and that the carrier mobility in the bulk is larger than that at the bottom surface at all temperatures. The pinning of the *E*_F_ at the BCB is found to be a key issue to induce the S-M transition, because the transition can be caused by depinning of the *E*_F_ or the crossover between the bulk and the top surface transport.

## Introduction

Bismuth selenide (Bi_2_Se_3_) is a typical three-dimensional topological insulator which has an insulating bulk state with a band gap and a conducting surface state with no band gap^1,2^. Electron spin at the surface state is helically polarized^3^, attracting much interest from the perspective of spintronics^4,5^. However, it is difficult to ascertain the surface conduction because the transport is dominated by bulk conduction owing to the natural deficiency of Se atoms and accumulation of excess electrons in the bulk region. One solution to observe the surface conduction is to replace trivalent Bi atoms with divalent or monovalent atoms. In fact, divalent Ca^6,7^ or Cd^8^ atoms work as *p* dopants when substituted for Bi atoms. Therefore, the Fermi level (*E*_F_) decreases from the bulk conduction band (BCB) to the bulk valence band (BVB). Impurity doping is used not only for tuning the *E*_F_, but also for achieving ordered states. Superconductivity by doping with Cu^9,10^, Sr^11,12^, or Nb^13,14^ atoms has been observed and discussed in relation to its topological nature. Furthermore, ferromagnetism by doping with Mn^15^, Cr^16^, or V^17^ atoms can lead to novel quantum phenomena, such as the quantum anomalous Hall effect. Thus, impurity doping allows to study a variety of electronic phenomena induced in topological insulators.

Recently, it was observed that a simple monovalent impurity, Ag, provided several intriguing phenomena^18,19^. First, bulk Ag-doped Bi_2_Se_3_ exhibited pressure-induced superconductivity, indicating the possibility of *p*-wave superconductivity^18^. Second, ultrathin Ag-doped Bi_2_Se_3_ showed a semiconductor–metal (S-M) transition with decreasing temperature^19^. The semiconducting behavior at high temperature is explained by the decrease in electron density owing to the substitution of Ag for Bi atoms. With decreasing temperature, resistance abruptly dropped at the transition temperature *T*_cr_ of approximately 35 K. Recently, a similar temperature dependence of resistance was reported in Cu-doped Bi_2_Se_3_ by two groups^20,21^. The following phenomena are commonly observed in the Cu-doped and Ag-doped Bi_2_Se_3_ ultrathin flakes. (i) *T*_cr_ was approximately 37 K^20^ or 30 K^21^ for Cu-doped Bi_2_Se_3_, similar to 35 K^19^ for Ag-doped Bi_2_Se_3_. (ii) Hysteresis in resistance during the cooling and heating processes was observed below *T*_cr_ in Cu-doped Bi_2_Se_3_^20^ and Ag-doped Bi_2_Se_3_ as described later. Thus, the results of this study of the S-M transition in Ag-doped Bi_2_Se_3_ may not be characteristic of Ag impurity however a universal phenomenon in doped Bi_2_Se_3_. Although the origin of the transition is unknown, hybridization between Cu^+^ and Cu^2+^ conduction bands or the crossover of two types of carrier transport has been proposed in Cu-doped Bi_2_Se_3_. In this study, extended analyses of Hall effect and electric-field effect are carried out to investigate the origin of the anomalous S-M transition observed in Ag-doped Bi_2_Se_3_. Ag impurities can donate or accept electrons, based on the impurity sites, interstitial site through intercalation or substitution of the Bi site, respectively. Because of the dual doping effects, *E*_F_ is fixed around the bottom of the BCB. This level of the *E*_F_ plays a key role to induce the S-M transition.

## Results and discussion

### S-M transition in Ag_***x***_Bi_2_Se_3_

**Figure 1:**
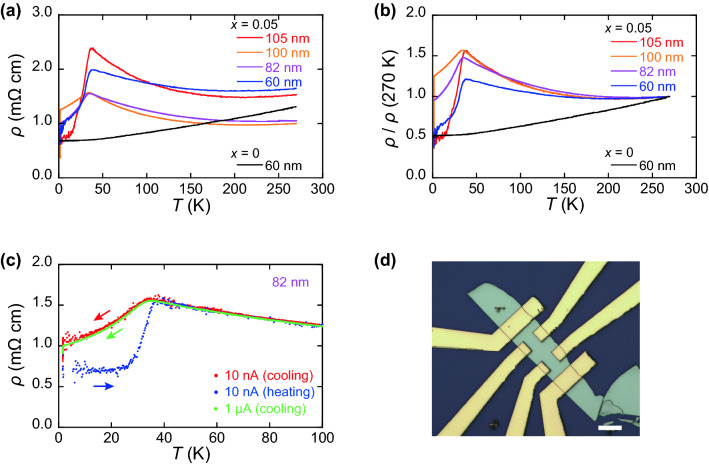
Temperature dependence of (**a**) resistivity and (**b**) normalized resistivity at 270 K of Ag_*x*_Bi_2_Se_3_ (*x* = 0.05 and 0) with various thicknesses. Resistivity is measured in the cooling process. The data from devices of 105 and 60 nm thicknesses were used in our previous report^19^. (**c**) Hysteretic behavior of resistivity observed in Ag_0.05_Bi_2_Se_3_ with 82 nm thickness. Resistivity measured during the cooling and heating processes is indicated by the directions of arrows. (**d**) Optical microscope image of a sample with 100 nm thickness. Scale bar indicates 10 μm.

Figure 1a–c summarize the S-M transition observed in Ag_*x*_Bi_2_Se_3_, and Fig. 1d indicates an optical microscope image of a typical device. Figure 1a shows the temperature dependence of the resistivity of Ag_0.05_Bi_2_Se_3_ and Bi_2_Se_3_ with various thicknesses. Bi_2_Se_3_ exhibits metallic behavior; the resistivity monotonically decreases with decreasing temperature. On the contrary, the resistivity of all Ag_0.05_Bi_2_Se_3_ devices increased with decreasing temperature and decreased below *T*_cr_ = 35 K. The resistivity shown in Fig. 1a does not exhibit a systematic thickness dependence. However, when it is normalized by the resistivity at 270 K, clear thickness dependence appears in the semiconducting region above 35 K. As shown in Fig. 1b, the increase in the normalized resistivity above *T*_cr_ becomes prominent when the thickness increases. This implies that the semiconducting behavior is caused by the bulk properties of the samples because the influence of the metallic surface state on the total transport property diminishes with increasing the thickness.

The $\rho \left(T\right)$ curve of Ag_*x*_Bi_2_Se_3_ was hysteretic below *T*_cr_, that is, $\rho \left(T\right)$ was different during the cooling or heating process. A device with a thickness of 82 nm, as shown in Fig. 1c, exhibits an example of the hysteretic behavior. The device was cooled from *T* = 270 to 1.6 K at a rate of 0.50 K/min, maintained at 1.6 K for 2 h, and heated to 0.89 K/min. The resistivity traces different paths below *T*_cr_, although it is identical above *T*_cr_. Because the resistivity measured at a maximum bias current of 1 μA (green symbol) is the same as that measured at 10 nA (red and blue symbols), the hysteresis is not caused by heating of the sample. Thus, hysteresis is not an experimental artifact caused by the disagreement of temperatures between the sample and the environment. This hysteretic behavior indicates a gradual decrease in resistivity below *T*_cr_, because extended time is required to establish an equilibrium electronic state. In fact, the resistance gradually decreased at fixed temperature below *T*_cr_ as shown in Fig. S1 in the supplementary information.

### Absence of structural transition

**Figure 2:**
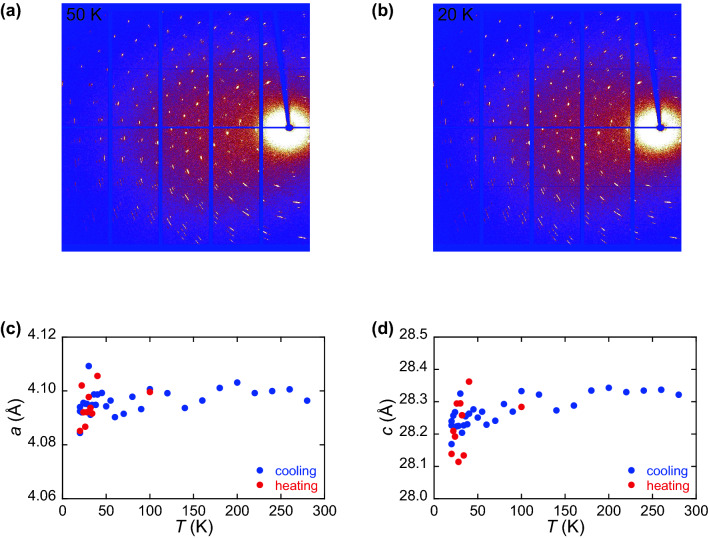
SC-XRD patterns obtained at (**a**) 50 K and (**b**) 20 K. Temperature dependence of lattice parameters of Ag_0.05_Bi_2_Se_3_, (**c**) *a* and (**d**) *c*, determined by SC-XRD. Blue and red symbols indicate the data measured in cooling and heating processes, respectively.

Single-crystal X-ray diffraction (SC-XRD) measurement was performed to explore the possibility that the transition has a structural origin, because some layered materials exhibit CDW transition or lattice distortion^22–24^, which can change the transport property. Figure 2a,b show the SC-XRD patterns measured at 50 and 20 K, i.e., above and below *T*_cr_ = 35 K, respectively. As shown in the figure, the two patterns show no significant difference. Therefore, the lattice parameters were calculated using the same rhombohedral structure of the space group: $R\overline{3 }m$, which has the same structure as non-doped Bi_2_Se_3_. Figure 2c,d show the temperature dependence of the lattice parameters *a* and *c* measured during the cooling and heating processes. Within the scattering of the data, neither CDW superlattice nor significant change in the lattice parameters was observed at *T*_cr_, however a smooth decrease in the two parameters was observed as temperature decreased.

Our previous experiment of Ag-doped Bi_2_Se_3_ under the pressure showed that the application of pressure induced the superconductivity owing to the structural transition at 8.8 GPa^18^. With increasing pressure from 0.18 to 8.8 GPa, the lattice parameters *a* and *c* decreased from 4.12 to 3.95 Å and from 28.6 to 27.2 Å, respectively, in which the variation of *a* and *c* were ∆*a* =  − 0.17 Å and ∆*c* =  − 1.4 Å. In this pressure range, the resistance increased by a factor of 4 with decreasing *a* and *c*. If the lattice shrinkage at low temperatures causes the same effect as physical pressure, the resistance should increase at low temperatures. Furthermore, the variation in *a* and *c* with decreasing temperature shown in Fig. 2 are ∆*a* ~  − 0.01 Å and ∆*c* ~  − 0.2 Å, which are much smaller than those with increasing pressure. Therefore, the abrupt change in the transport property at *T*_cr_ is ascribed to neither the structural transition nor the lattice shrinkage, assuming that the same order of variation of *a* and *c* is expected for the SM transition and the superconducting transition. A more detailed analysis of the crystal structure was difficult because of the twin structure of Ag-doped Bi_2_Se_3_, in which the twinning axis *b* is shared.

### Increase in carrier density and Hall mobility below *T*_cr_

**Figure 3:**
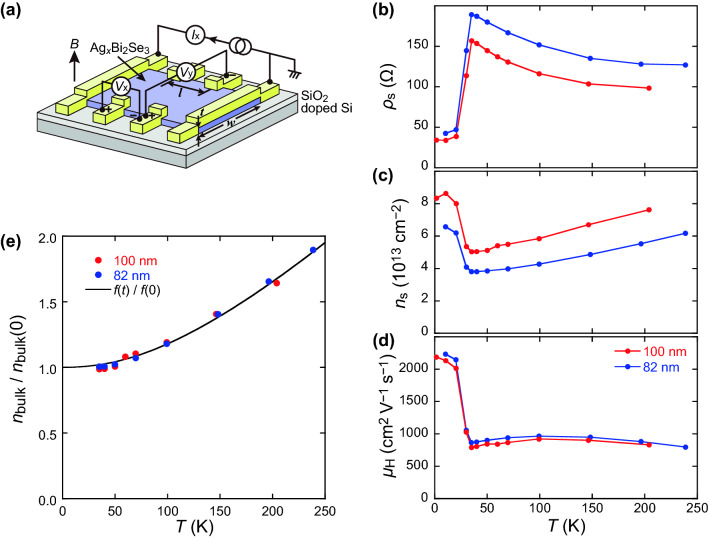
(**a**) Schematic configuration for Hall effect measurement. Temperature dependence of (**b**) sheet resistance, (**c**) electron density per unit area, and (**d**) Hall mobility. Red and blue data were obtained from the devices with 100 and 82 nm thickness, respectively. The data in (**c**) from the device with 82 nm thickness was used in our previous report^19^. (**e**) Temperature dependence of bulk carrier density normalized by the value at zero temperature. The data at the semiconducting state ($T\ge$ 35 K) are shown.

Experimental setup for the Hall effect measurement is shown in Fig. 3a. The transverse voltage *V*_y_ was measured to obtain *R*_yx_ (= *V*_y_/*I*_x_). The carrier density *n*_s_ and Hall mobility *μ*_H_ was analyzed from *R*_yx_, which is linear to the magnetic field in the range from − 1 to 1 T (see Fig. S2 in the supplementary information). Figure 3b–d show the temperature dependence of *ρ*_s_, *n*_s_, and *μ*_H_, respectively, obtained from two devices, #1 and #2. The thicknesses of the Ag_*x*_Bi_2_Se_3_ flakes were 82 and 100 nm for #1 and #2, respectively. Part of the data from #1 was used in our previous study^19^. The measurements were performed at the heating process after waiting enough time at the lowest temperature. The S-M transition at *T*_cr_ = 35 K was reproduced in two devices, as shown in Fig. 3b. Figure 3c,d show that decrease in *ρ*_s_ below *T*_cr_ is accompanied by an increase in both the carrier concentration and Hall mobility. As shown in Fig. 3c, the carrier concentration decreases with decreasing temperature, whereas it increases below *T*_cr_. Above *T*_cr_, *n*_s_ decreases almost linearly with decreasing temperature, indicating that the carrier is thermally excited and *E*_F_ is located near the bottom of the BCB. The minimum carrier concentration at *T*_cr_, $3.8\times {10}^{13} \;\mathrm{ c}{\mathrm{m}}^{-2}$, exceeds the carrier concentration at the surface states of Bi_2_Se_3_, $1\times {10}^{13} \; \mathrm{ c}{\mathrm{m}}^{-2}$^25,26^. Thus, the dominant carrier arises from the bulk rather than from the surface alone. On the contrary, the Hall mobility is almost constant above *T*_cr_, whereas it abruptly increases below *T*_cr_, as shown in Fig. 3d.

### Position of the Fermi level

Here, the Fermi level was evaluated from the temperature dependence of the carrier density per unit area ${n}_{\mathrm{s}}(T)$ shown in Fig. 3c. The total carrier density ${n}_{\mathrm{s}}(T)$ consists of carriers at the surface ${n}_{\mathrm{s}(\mathrm{surf})}$ and those in the bulk ${n}_{\mathrm{s}(\mathrm{bulk})}$, in which only ${n}_{\mathrm{s}(\mathrm{bulk})}$ is assumed to depend on $T$; ${n}_{\mathrm{s}}\left(T\right)={n}_{\mathrm{s}(\mathrm{surf})}+{n}_{\mathrm{s}(\mathrm{bulk})}(T)$. The value of ${n}_{\mathrm{s}(\mathrm{bulk})}(T)$ is expressed as follows:

1 $${n}_{\mathrm{s}(\mathrm{bulk})}\left(T\right)={\int }_{0}^{\infty }\frac{D(E)dE}{\mathrm{exp}\left[\left(E-{E}_{\mathrm{F}}\right)/{k}_{\mathrm{B}}T\right]+1} ,$$

where $E$ is measured from the BCB minimum, and $D(E)\equiv a{E}^{1/2}$ represents the density of states based on the 3-dimensional free electron model. Substituting $\varepsilon =E/{E}_{\mathrm{F}}$ and *t* $={k}_{\mathrm{B}}T/{E}_{\mathrm{F}}$, ${n}_{\mathrm{s}(\mathrm{bulk})}(T)$ can be simplified as follows:

2 $$\begin{aligned} {n}_{\mathrm{s}(\mathrm{bulk})}\left(T\right) & =a{E}_{\mathrm{F}}^\frac{3}{2}{\int }_{0}^{\infty }\frac{{\varepsilon }^\frac{1}{2}d\varepsilon }{\mathrm{exp}\left[\frac{\varepsilon -1}{t}\right]+1} \\ & \equiv a{E}_{\mathrm{F}}^\frac{3}{2}f\left(t\right).\end{aligned}$$

Thus, when ${n}_{\mathrm{s}(\mathrm{bulk})}\left(T\right)$ is normalized by ${n}_{\mathrm{s}(\mathrm{bulk})}\left(0\right)$, it is expressed by the universal function $f\left(t\right)/f\left(0\right)$. Two datasets of ${n}_{\mathrm{s}}\left(T\right)$ for 82 and 100 nm thick crystals in Fig. 3c were transformed by adjusting the parameter ${n}_{\mathrm{s}(\mathrm{surf})}$, so that the values

3 $$\frac{{n}_{\mathrm{s}}\left(T\right)-{n}_{\mathrm{s}(\mathrm{surf})}}{{n}_{\mathrm{s}}\left(0\right)-{n}_{\mathrm{s}(\mathrm{surf})}}\left(=\frac{{n}_{\mathrm{s}(\mathrm{bulk})}\left(T\right)}{{n}_{\mathrm{s}(\mathrm{bulk})}\left(0\right)}=\frac{f\left(t\right)}{f\left(0\right)}\right)$$

show the same temperature dependence above $T>{T}_{\mathrm{cr}}$. The values of ${n}_{\mathrm{s}}\left(0\right)$ were evaluated by extrapolating each ${n}_{\mathrm{s}}\left(T\right)$ curve at $T>{T}_{\mathrm{cr}}$ to $T=0$. Using two fitting parameters, ${n}_{\mathrm{s}(\mathrm{surf})}=1.13\times {10}^{13} \; {\mathrm{cm}}^{-2}$ and ${E}_{\mathrm{F}}=23.5 \;\; \mathrm{meV}$, the two data sets of ${n}_{\mathrm{s}}\left(T\right)$ in Fig. 3c were made to fall on the common black curve, $f\left(t\right)/f\left(0\right)$, as shown in Fig. 3e. The above value of ${n}_{\mathrm{s}(\mathrm{surf})}$ agrees fairly well with the literature value of the maximum electron density at the surface states, $1\times {10}^{13} \; {\mathrm{cm}}^{-2}$^25,26^, indicating the validity of this model. Furthermore, the small ${E}_{\mathrm{F}}$ of 23.5 meV evaluated by the above model indicates that the ${E}_{\mathrm{F}}$ is located immediately above the BCB minimum at $T>{T}_{\mathrm{cr}}$.

In this section, we discussed temperature dependence of *n*_s_ shown in Fig. 3c. Furthermore, we can compare temperature dependence of the sheet conductivity *σ*_s_ of the two samples using Fig. 3b. The difference of the sheet conductivity ∆*σ*_s_ approximately gives the bulk conduction alone, which increases below *T*_cr_ as shown in Fig. S3a in the supplementary information.

### Different field-effect mobility at positive and negative gate voltages

The carrier mobility was also evaluated from electric-field effect measurements to distinguish between bulk and surface conduction. The Hall effect reflects the entire conduction in the bulk and the surface of the material, whereas the electric field effect is more sensitive to surface conduction. This is because the carrier density only at the bottom surface is modulated by *V*_g_. Based on the two-carrier model, the total conductivity consists of the bulk and the two surfaces at top and bottom: ${\sigma }_{\mathrm{s}}={\sigma }_{\mathrm{s}(\mathrm{top})}+{\sigma }_{\mathrm{s}(\mathrm{bulk})}+{\sigma }_{\mathrm{s}(\mathrm{bottom})}$. Here, the conductivity is the product of carrier density and mobility: ${\sigma }_{\mathrm{s}\left(i\right)}={n}_{\mathrm{s}(i)}e{\mu }_{(i)}$ (*i* = top, bulk, or bottom). When applying *V*_g_, the ${\sigma }_{\mathrm{s}}$ changes as $\frac{d{\sigma }_{\mathrm{s}}}{d{V}_{\mathrm{g}}}=\frac{d{\sigma }_{\mathrm{s}(\mathrm{top})}}{d{V}_{\mathrm{g}}}+\frac{d{\sigma }_{\mathrm{s}(\mathrm{bulk})}}{d{V}_{\mathrm{g}}}+\frac{d{\sigma }_{\mathrm{s}(\mathrm{bottom})}}{d{V}_{\mathrm{g}}}\sim \frac{d{\sigma }_{\mathrm{s}(\mathrm{bottom})}}{d{V}_{\mathrm{g}}}={C}_{\mathrm{o}}{\mu }_{(\mathrm{bottom})}$, where ${C}_{\mathrm{o}}(=11.5 \; \mathrm{ nF } \; {\mathrm{cm}}^{-2})$ represents the capacitance per unit area for a gate insulator of SiO_2_ 300 nm thick. Thus, we can discuss ${\mu }_{(\mathrm{bottom})}$ from $\frac{d{\sigma }_{\mathrm{s}}}{d{V}_{\mathrm{g}}}$ regardless of the magnitude of the total ${\sigma }_{\mathrm{s}}$.

**Figure 4:**
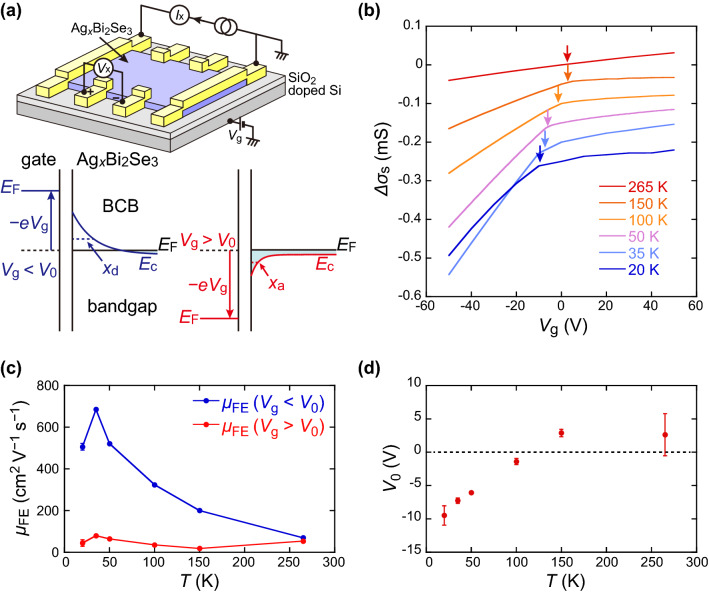
(**a**) Schematic configuration for field-effect measurement, and band diagram at the interface between gate electrode/gate insulator/Ag_*x*_Bi_2_Se_3_. The Fermi energy *E*_F_ of Ag_*x*_Bi_2_Se_3_ is assumed to be located immediately above the bottom of BCB. The conduction band minimum of Ag_*x*_Bi_2_Se_3_, *E*_c_, under negative and positive *V*_g_ is indicated with blue and red lines, respectively. Note that depletion depth *x*_d_ is much larger than accumulation depth *x*_a_. (**b**) Gate voltage dependence of sheet conductivity of sample #2 at various temperatures. The curves are offset for clarity. The arrows indicate the inflection points. (**c**) Temperature dependence of field-effect mobility evaluated from *V*_g_ < *V*_0_ (blue line) and *V*_g_ > *V*_0_ (red line). (**d**) Temperature dependence of the inflection point, *V*_0_.

Experimental setup for the electric-field effect measurement is shown in Fig. 4a. The ${\sigma }_{\mathrm{s}}({V}_{\mathrm{g}})$ curves at different temperatures for #2 are shown in Fig. 4b. The curves are offset for clarity, so that the values of ${\sigma }_{\mathrm{s}}(0)$ are shifted by 0.05 mS. The inverses of ${\sigma }_{\mathrm{s}}(0)$ (or ${\rho }_{\mathrm{s}}(0)$) are indicated as a function of *T*, as shown in Fig. 3b. The slope of all ${\sigma }_{\mathrm{s}}({V}_{\mathrm{g}})$ curves, $\mathrm{d}{\sigma }_{\mathrm{s}}/{\mathrm{d}V}_{\mathrm{g}}$, is positive, indicating that the charge carrier is *n*-type, which is in agreement with the result of the Hall effect. In this study, the inflection of the curves around the zero gate voltage is noticed. Because the slope of ${\sigma }_{\mathrm{s}}({V}_{\mathrm{g}})$ curve is proportional to the field-effect mobility, ${\mu }_{\mathrm{FE}}$, the inflection of the curve implies that ${\mu }_{\mathrm{FE}}$ is larger at ${V}_{\mathrm{g}}<{V}_{0}$ than at ${V}_{\mathrm{g}}>{V}_{0}$, where ${V}_{0}$ is an inflection point. The ${V}_{\mathrm{g}}$-dependent mobility is responsible for Ag impurities because such a peculiar ${\sigma }_{\mathrm{s}}({V}_{\mathrm{g}})$ curve has not been reported in non-doped Bi_2_Se_3_. To evaluate the data quantitatively, they were fitted using the following equation

4 $${\sigma }_{\mathrm{s}}\left({V}_{\mathrm{g}}\right)={-a}_{1}\left|{V}_{\mathrm{g}}-{V}_{0}\right|+{a}_{2}{V}_{\mathrm{g}}+{a}_{3},$$

where *a*_*i*_ (*i* = 1…3), and *V*_0_ represent the fitting parameters. The values of ${\mu }_{\mathrm{FE}}$ were evaluated by ${{\mu }_{\mathrm{FE}}^{{(V}_{\mathrm{g}} < {V}_{0})}\equiv (a}_{1}+{a}_{2})/{C}_{\mathrm{o}}$ at ${V}_{\mathrm{g}}<{V}_{0}$ and ${{\mu }_{\mathrm{FE}}^{{(V}_{\mathrm{g}} > {V}_{0})}\equiv (-a}_{1}+{a}_{2})/{C}_{\mathrm{o}}$ at ${V}_{\mathrm{g}}>{V}_{0}$. The temperature dependences of ${\mu }_{\mathrm{FE}}$ and ${V}_{0}$ are shown in Fig. 4c,d, respectively. Note that ${\mu }_{\mathrm{FE}}^{{(V}_{\mathrm{g}} < {V}_{0})}$ is larger than ${\mu }_{\mathrm{FE}}^{{(V}_{\mathrm{g}} > {V}_{0})}$ over the entire temperature range. In particular, ${\mu }_{\mathrm{FE}}^{{(V}_{\mathrm{g}} < {V}_{0})}$ significantly increases with decreasing temperature above ${T}_{\mathrm{cr}}$, which is in contrast to the almost constant ${\mu }_{\mathrm{FE}}^{{(V}_{\mathrm{g}} > {V}_{0})}$ and ${\mu }_{\mathrm{H}}$ above ${T}_{\mathrm{cr}}$. The inflection point *V*_0_ changed from positive to negative with decreasing temperature. As discussed later, the results are explained by the different mobilities at the surface and bulk states.

### Difference between three kinds of mobilities

When *E*_F_ is adjacent to the BCB minimum, the gate voltage has a significant impact on the electronic state, which can mean the difference between ${\mu }_{\mathrm{FE}}^{{(V}_{\mathrm{g}} < {V}_{0})}$ and ${\mu }_{\mathrm{FE}}^{{(V}_{\mathrm{g}} > {V}_{0})}$. As shown in the band diagrams in Fig. 4a, electrons with sheet density of $n\left(\equiv -{C}_{\mathrm{o}}{V}_{\mathrm{g}}/e\right)$ are removed by applying a negative *V*_g_, whereas those of $n\left(\equiv {C}_{\mathrm{o}}{V}_{\mathrm{g}}/e\right)$ accumulated by applying a positive *V*_g_. In general, the depletion depth *x*_d_ is much longer than the accumulation depth *x*_a_^27^. The existence of the surface state may narrow the difference between ${x}_{\mathrm{d}}$ and ${x}_{\mathrm{a}}$. However, because the surface state is too thin to shield the electric field completely, the relation of ${x}_{\mathrm{d}}>{x}_{\mathrm{a}}$ will still hold.

To explain the different field-effect mobility at ${V}_{\mathrm{g}}<{V}_{0}$ and ${V}_{\mathrm{g}}>{V}_{0}$, a two-layer model is considered; the surface layer of thickness ${d}_{\mathrm{s}}$ is related to the surface mobility of ${\mu }_{\mathrm{s}}\left(={\mu }_{(\mathrm{bottom})}\right)$, and the other region to the bulk mobility of ${\mu }_{\mathrm{b}} (={\mu }_{\left(\mathrm{bulk}\right)})$. Assuming the length relation ${x}_{\mathrm{a}}<{d}_{\mathrm{s}}<{x}_{\mathrm{d}}$, the sheet conductivity approximately changes with *V*_g_ as follows:

5 $$\begin{aligned} {\sigma }_{\mathrm{s}}^{\left({V}_{\mathrm{g}} < {V}_{0}\right)}\left({V}_{\mathrm{g}}\right)& ={\sigma }_{\mathrm{s}}\left(0\right)-\left(n\frac{{d}_{\mathrm{s}}}{{x}_{\mathrm{d}}}\right)e{\mu }_{\mathrm{s}}-\left(n\frac{{x}_{\mathrm{d}}-{d}_{\mathrm{s}}}{{x}_{\mathrm{d}}}\right)e{\mu }_{\mathrm{b}} \\ & ={\sigma }_{\mathrm{s}}\left(0\right)+\left\{\frac{{d}_{\mathrm{s}}}{{x}_{\mathrm{d}}}{\mu }_{\mathrm{s}}+\left(1-\frac{{d}_{\mathrm{s}}}{{x}_{\mathrm{d}}}\right){\mu }_{\mathrm{b}}\right\}{C}_{\mathrm{o}}{V}_{\mathrm{g}} \;\; \text{at} \;\; {V}_{\mathrm{g}}<{V}_{0}, \;\; \mathrm{ and} \;\; \end{aligned}$$

6 $${\sigma }_{\mathrm{s}}^{\left({V}_{\mathrm{g}} > {V}_{0}\right)}\left({V}_{\mathrm{g}}\right)={\sigma }_{\mathrm{s}}\left(0\right)+ne{\mu }_{\mathrm{s}}={\sigma }_{\mathrm{s}}\left(0\right)+{\mu }_{\mathrm{s}}{C}_{\mathrm{o}}{V}_{\mathrm{g}} \;\; \text{at} \;\; {V}_{\mathrm{g}}>{V}_{0}.$$

Here, the effective capacitance at the interface may be smaller than *C*_o_ at *V*_g_ < *V*_0_ due to the capacitance of the depletion layer connected in series^27^. This effect was neglected for simplicity, because the reduction of the effective capacitance decreases the slope of the $\sigma \left({V}_{\mathrm{g}}\right)$ curves at *V*_g_ < *V*_0_, which is opposite to the experimental result. From Eqs. (5) and (6), the field-effect mobility ${\mu }_{\mathrm{FE}}\left(\equiv \frac{1}{{C}_{o}}\left|\frac{d\sigma }{d{V}_{\mathrm{g}}}\right|\right)$ is given by

7 $${\mu }_{\mathrm{FE}}^{{(V}_{\mathrm{g}} <{ V}_{0})}=\frac{{d}_{\mathrm{s}}}{{x}_{\mathrm{d}}}{\mu }_{\mathrm{s}}+\left(1-\frac{{d}_{\mathrm{s}}}{{x}_{\mathrm{d}}}\right){\mu }_{\mathrm{b}} \;\; \text{at} \;\; {V}_{\mathrm{g}}<{V}_{0}, \;\; \text{and}$$

8 $${\mu }_{\mathrm{FE}}^{{(V}_{\mathrm{g}} > {V}_{0})}={\mu }_{\mathrm{s}} \;\; \text{at} \;\; {V}_{\mathrm{g}}>{V}_{0}.$$

As shown in Eqs. (7) and (8), ${\mu }_{\mathrm{FE}}^{{(V}_{\mathrm{g}} < {V}_{0})}$ obtained by removing the electrons within the depletion depth provides helpful information on the mobility in the bulk, compared with ${\mu }_{\mathrm{FE}}^{{(V}_{\mathrm{g}} > {V}_{0})}$ evaluated in the accumulation region. The fact that ${\mu }_{\mathrm{FE}}^{{(V}_{\mathrm{g}} < {V}_{0})}> {\mu }_{\mathrm{FE}}^{{(V}_{\mathrm{g}} > {V}_{0})}$ indicates that the electron mobility is higher in the bulk than at the surface. This conclusion is also supported by the comparison between ${\mu }_{\mathrm{H}}$ and ${\mu }_{\mathrm{FE}}$. As shown in Figs. 3d and 4c, an inequality, ${\mu }_{\mathrm{H}}>{\mu }_{\mathrm{FE}}^{{(V}_{\mathrm{g}} < {V}_{0})}> {\mu }_{\mathrm{FE}}^{{(V}_{\mathrm{g}} > {V}_{0})}$ holds at each temperature. This magnitude relation between three mobilities shows that the bulk mobility is larger than the surface mobility because ${\mu }_{\mathrm{H}}$ and ${\mu }_{\mathrm{FE}}$ reflect the conduction in the entire film and at the surface, respectively. In addition, it has been reported that the surface mobility is lower than the bulk mobility in undoped Bi_2_Se_3_^28^.

Owing to the assumption that ${\mu }_{\mathrm{b}}>{\mu }_{\mathrm{s}}$, ${\mu }_{\mathrm{FE}}^{{(V}_{\mathrm{g}} <{ V}_{0})}$ is always larger than ${\mu }_{\mathrm{FE}}^{{(V}_{\mathrm{g}} > {V}_{0})}$. Furthermore, this simple model can explain not only the different field-effect mobilities, but also the temperature dependence of ${\mu }_{\mathrm{FE}}^{{(V}_{\mathrm{g}} < {V}_{0})}$ shown in Fig. 4c. With decreasing temperature, ${x}_{\mathrm{d}}$ is enhanced because the thermally excited carrier density ${n}_{\mathrm{s}}$ decreases^24^. As a result, ${\mu }_{\mathrm{FE}}^{{(V}_{\mathrm{g}} < {V}_{0})}$ approaches ${\mu }_{\mathrm{b}}$ from Eq. (7) as shown in Fig. 4c. The slight decrease in ${\mu }_{\mathrm{FE}}^{{(V}_{\mathrm{g}} < {V}_{0})}$ below *T*_cr_ (from 35 to 20 K, see Fig. 4c), which is contrary to the behavior of ${\mu }_{\mathrm{H}}$ in Fig. 3d, is qualitatively explained by the increase in the carrier density *n*_s_ indicated in Fig. 3c. Then, ${x}_{\mathrm{d}}$ decreases in turn, and ${\mu }_{\mathrm{FE}}^{{(V}_{\mathrm{g}} <{ V}_{0})}$ decreases according to Eq. (7).

Finally, we discuss the possibility that the SM transition is induced by the band bending at the interface. The inflection point ${V}_{0}$, which separates the depletion and accumulation regions, is the gate voltage required to flatten the band at the interface (see Fig. 4a). The electron density accumulated at the bottom surface without *V*_g_ was estimated as ${{n}_{0}\equiv -C}_{\mathrm{o}}{V}_{0}/e$ for negative ${V}_{0}$. As shown in Fig. 4d, ${V}_{0}$ smoothly varies from + 2.5 to − 10 V with decreasing temperature. That is, the temperature dependence of ${n}_{0}$ has no singularity at *T*_cr_. The increase in ${n}_{0}$ from 300 to 25 K is maximum $9\times {10}^{11} \; {\mathrm{cm}}^{-2}$, which is much smaller than that of ${n}_{\mathrm{s}}$ $(>2\times {10}^{13} \; {\mathrm{ cm}}^{-2})$ shown in Fig. 3c. Thus, the change in the surface carrier due to the band bending does not affect the transition at *T*_cr_, which suggests that the bottom surface is not related to the transition.

### Roles of Ag atoms for carrier doping

In this section, the roles of Ag dopant in Ag_*x*_Bi_2_Se_3_ for the doping of the carriers and the level of the *E*_F_ are discussed. The *E*_F_ in Ag_*x*_Bi_2_Se_3_ is fixed at around the bottom of the BCB despite of *x* in the range from 0.05 to 0.2^19^, which we call the pinning of *E*_F_. To pin the *E*_F_ at the same level, the carrier density must be fixed even if the Ag impurity atoms induce carriers in Bi_2_Se_3_. When a monovalent Ag atom is substituted for a trivalent Bi atom, two holes are accumulated, or two excess electrons are compensated. On the contrary, when an Ag atom is intercalated into the van der Waals gap between the Se-Se layers, one electron is supplied from the Ag atom. When *x* is small, excess electrons in Bi_2_Se_3_ in the BCB can be removed by the substitution of Ag for Bi. The substitution of Bi with Ag was supported by the observation with X-ray fluorescence holography^18^. However, once the *E*_F_ reaches the bottom of the BCB (this can occur at *x* = 7.2 × 10^−4^, by assuming a typical electron density of 1 × 10^19^ cm^−3^ in Bi_2_Se_3_), further electrons must be removed from the BVB, requiring additional energy of 0.3 eV^2^ owing to the existence of the bulk band gap. The value of *x* = 0.05 in the sample of this study, is sufficient for the ${E}_{\mathrm{F}}$ to reach the bottom of the BCB. The fact that the carrier is *n*-type even at *x* = 0.05 suggests that a significant portion of Ag atoms are intercalated to provide an electron. Thus, the number of impurity sites occupied by Ag atoms may be determined so that the orbital energy of electrons in the BCB can be minimized. When the ratio of the substituted and intercalated Ag atoms is 1:2, the carrier density is preserved regardless of the value of *x*. The detailed balance between substitution and intercalation may pin the *E*_F_ around the bottom of the BCB. In fact, recently both substituted and intercalated Ag atoms have been observed with both X-ray photoemission and fluorescence holographies^29^.

### Possible origins for the S-M transition

Here, we present two possible mechanisms which can induce the S-M transition. One originates from the bulk state, and the other from the top surface sate. The obtained results from transport measurements are summarized as follows: (i) semiconducting behavior of ${\upsigma }_{\mathrm{s}}$ above *T*_cr_ is due to the bulk (Figs. 1b and S3a), (ii) increase in *n*_s_ below *T*_cr_ exceeds the surface carrier concentration. (Fig. 3c), (iii) carrier mobility is larger in the bulk than at the bottom at all temperatures (Fig. 4c), and (iv) temperature dependence of ${\upsigma }_{\mathrm{s}}$ is qualitatively similar to that of the bulk (Fig. S3a). These results suggest that the transition originates from the change in the bulk electronic state, but the possibility that the top surface contributes to ${\upsigma }_{\mathrm{s}}$ below *T*_cr_ cannot be excluded.

**Figure 5:**
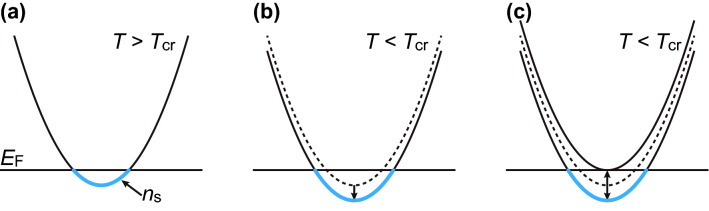
Possible scenarios to increase the carrier density at the S-M transition. (**a**) The *E*_F_ is pinned on the bottom of the BCB above *T*_cr_. The BCB is indicated by the parabolic line in which blue states under the *E*_F_ are occupied by the electrons. (**b**) The BCB is lowered owing to the expansion of the band width below *T*_cr_. (**c**) The BCB is split owing to electron correlation below *T*_cr_.

Assuming the pinning of the *E*_F_ at *T* > *T*_cr_, the S-M transition can be explained by the depinning of *E*_F_ and the increase in *n*_s_ at *T* < *T*_cr_, as shown in Fig. 3c. Figure 5 illustrates the scenarios which explain the depinning of *E*_F_. Figure 5b shows the change in the energy relation between the *E*_F_ and the bottom of the BCB. For example, the shrinkage of the crystal lattice at low temperatures may expand the bandwidth to lower the BCB under *E*_F_. However, the reduction of the lattice parameters under the pressure increased the resistance^18^, in contrast to our result. Band splitting owing to electron correlation below *T*_cr_ (see Fig. 5c) could also enhance *n*_s_. In this case, the increase in *n*_s_ comes from the split band with lower energy, and the increase in ${\mu }_{\mathrm{H}}$ is also possible in the correlated states. If band splitting is accompanied by spin correlation, it may take long time to establish the equilibrium state, which can explain the hysteretic behavior observed at low temperatures. The Rashba effect also could cause the spin–orbit band splitting, which has been observed at the surface of the Bi_2_Se_3_^30^. The change in the band structure is expected to be observed using the ARPES. The possibility of such novel electronic states will be further explored in the future.

Another mechanism is due to the crossover between the bulk and surface conduction^31,32^. There was no structural transition at *T*_cr_, and the transition was observed only in ultrathin samples^18^. These results support the surface effect on the transition, which becomes invisible in a thicker sample. One problem is that the field-effect mobility ${\mu }_{\mathrm{FE}}$ is lower than the Hall mobility ${\mu }_{\mathrm{H}}$ below *T*_cr_ as shown in Figs. 3d and 4c. This discrepancy can be explained by the different mobility at the top and bottom surfaces. The mobility at the top surface may be larger than the ${\mu }_{\mathrm{FE}}$ because ${\mu }_{\mathrm{FE}}$ can be limited by the interfacial scattering due to the substrate. If the mobility at the top surface layer exceeds that in the bulk below *T*_cr_, the transition to high ${\mu }_{\mathrm{H}}$ states is qualitatively explained by the conduction at the top surface. However, it should be noted that this is not simple crossover between the insulating bulk conduction and the metallic surface conduction, because the bulk conduction becomes metallic below *T*_cr_ as seen from Fig. S3a. The significant enhancement of the mobility at the top surface is required.

The two plausible mechanisms for high mobility can be examined by measuring the transport properties at high magnetic fields. The carrier density and the mobility in the bulk or the surface can be distinguished by analyzing the nonlinear Hall effect. Furthermore, if the top surface state gives the high mobility, the Shubnikov-de Haas oscillation will be observed in the resistivity^33,34^. The mechanism of the high mobility will be further studied in future.

## Conclusion

The transport properties of Ag-doped Bi_2_Se_3_ were studied under magnetic and electric fields. Based on the impurity sites, the Ag atom acts as a *p* or *n* dopant, causing the Fermi level to be fixed around the bottom of the BCB. The carrier number and carrier mobility are dominated by the bulk region rather than the surface state above *T*_cr_. Below *T*_cr_, the carrier density and mobility significantly increase, suggesting that the S-M transition is caused by the depinning of the *E*_F_ or the crossover between the bulk and surface conduction. The band splitting is suggested to be the possible origin of the depinning of the *E*_F_. The possibility of the surface conduction will be examined by transport measurement at the high magnetic field. These candidates will be investigated in the future. Ag doping is observed to be an effective method for probing the electronic state with low carrier concentration in topological insulators. In particular, the transition to a state with high mobility will attract interest from fundamental research and applications. The abrupt changes in the conductivity and mobility at the specific temperature add a new function to thermoelectric properties of Be_2_Se_3_. Furthermore, the inflection of ${\upsigma }_{\mathrm{s}}({V}_{\mathrm{g}})$ curve will be useful to sense the charge adsorbed at surface. In this way, doping effects on topological insulators are important also in development of novel functional devices.

In the same manner as the sample of this study, Ag_*x*_Bi_2_Se_3_, Cu_*x*_Bi_2_Se_3_ shows a similar S-M transition. Because of the small ionic radii of the impurity atoms, these materials have two kinds of impurity sites, substituted and intercalated. Note that these different impurity sites have also been observed in Sr_*x*_Bi_2_Se_3_^11,12^. If the pinning of ${E}_{\mathrm{F}}$ owing to the different roles of impurities is essential for the S-M transition, the same S-M transition would be anticipated in Sr_*x*_Bi_2_Se_3_. By tuning the ${E}_{\mathrm{F}}$, the doping method based on dual roles of impurities may pave the way for novel functionalization of topological materials.

## Methods

Bulk crystals of Ag_*x*_Bi_2_Se_3_ were grown using a melt-growth method described elsewhere^18,19^. The samples were prepared from the same bulk crystal used in our previous report^19^. In this study, the samples are denoted as Ag_0.05_Bi_2_Se_3_ based on the nominal molar ratio of Ag, Bi, and Se mixed. The bulk crystal was exfoliated with adhesive tape, and the ultrathin crystals were transferred onto a SiO_2_/Si substrate. Using the ultrathin Ag_*x*_Bi_2_Se_3_ crystal as a channel material, multi-terminal field-effect transistors (FETs) were prepared. Six electrodes (Cr 5 nm/Au 150–200 nm) were attached to the crystal in the Hall bar geometry as shown in Fig. 1d, and a gate electrode was connected to the Si substrate, allowing a bottom gate voltage *V*_g_ to be applied to the crystal through a SiO_2_ gate insulator. The device was cooled in a ^4^He cryostat using an Oxford superconducting magnet system. The temperature and magnetic field were controlled using mercury iTC and iPS, respectively. For the Hall effect measurement, longitudinal and transverse voltages (*V*_x_ and *V*_y_) were measured by supplying electric current *I*_x_. The current was supplied with a Keithley 220 programmable current source and measured using an Advantest R8240 digital electrometer. The voltage was measured using an Agilent 34420A nanovoltmeter. The longitudinal and transverse resistances (*R*_xx_ and *R*_yx_) were determined from d*V*_x_/d*I*_x_ and d*V*_y_/d*I*_x_, respectively. The resistivity $\rho$, sheet resistance ${\rho }_{\mathrm{s}}$, and sheet conductance ${\sigma }_{\mathrm{s}}$ were evaluated by $\rho \equiv {R}_{\mathrm{xx}}wt/l$, ${\rho }_{\mathrm{s}}\equiv \rho /t$, and ${\sigma }_{\mathrm{s}}\equiv 1/{\rho }_{\mathrm{s}}$, where $l$, $w$, and $t$ represent the channel length, width, and thickness, respectively. The transverse resistance ${R}_{\mathrm{yx}}$ was measured with respect to the perpendicular magnetic field *B*, and the Hall coefficient ${R}_{\mathrm{H}}$ was calculated as ${R}_{\mathrm{H}}\equiv t\frac{d{R}_{\mathrm{yx}}}{dB}$. *R*_H_ was negative when the charge carrier was an electron. The electron density per unit area, *n*_s_, and Hall mobility, ${\mu }_{\mathrm{H}}$, were evaluated as ${n}_{\mathrm{s}}=-\frac{t}{{eR}_{\mathrm{H}}}$ and ${\mu }_{\mathrm{H}}\equiv {\sigma }_{\mathrm{s}}\left|{R}_{\mathrm{H}}\right|/t$, respectively.

The electric-field effect was studied by applying *V*_g_. The field-effect mobility ${\mu }_{\mathrm{FE}}$ was calculated as ${\mu }_{\mathrm{FE}}\equiv \frac{1}{{C}_{\mathrm{o}}}\left|\frac{d{\sigma }_{\mathrm{s}}}{d{V}_{\mathrm{g}}}\right|$. The gate voltage was applied using a Keithley 2635A system source meter. After the transport measurements, the thickness of Ag_*x*_Bi_2_Se_3_ was measured using atomic force microscopy (SII technology, SPA400-DFM). To examine the possibility of the structural transition at *T*_cr_, single-crystal X-ray diffraction (SC-XRD) of Ag_0.05_Bi_2_Se_3_ was measured down to 20 K by blowing ^4^He gas, using synchrotron radiation at BL02B1 of SPring-8. The wavelength was 0.300900 Å.

## Supplementary Information

Supplementary Information.

## Data Availability

The datasets used and/or analysed during the current study are available from the corresponding author on reasonable request.
