# Supplementary material for: Semiconductor–metal transition in Bi2Se3 caused by impurity doping
Source: Sci Rep. 2023 Jan 11;13:537. doi: 10.1038/s41598-023-27701-5 (PMC9834400; doi:10.1038/s41598-023-27701-5)
Supplement: Supplementary file 1 — Supplementary Information. [file 41598_2023_27701_MOESM1_ESM.docx]

**Supplementary Information**

Semiconductor-metal transition in Bi_2_Se_3_ caused by impurity doping

Takaki Uchiyama^1^, Hidenori Goto^1,*^, Eri Uesugi^1^, Akihisa Takai^1^, Lei Zhi^1^, Akari Miura^1^, Shino Hamao^1^, Ritsuko Eguchi^1^, Hiromi Ota^2^, Kunihisa Sugimoto^3^, Akihiko Fujiwara^4^, Fumihiko Matsui^5^, Koji Kimura^6^, Kouichi Hayashi^6^, Teppei Ueno^1^, Kaya Kobayashi^1^, Jun Akimitsu^1^, Yoshihiro Kubozono^1^

Contents

1. Gradual reduction of resistance below *T*_cr_

2. Hall effect at low magnetic field.

3. Semi-quantitative analysis for bulk and surface conduction

1. Gradual reduction of resistance below *T*_cr_

The resistance was continuously measured at lowest temperature, 1.6 K. Figure S1 shows the resistance as a function of elapsed time. The resistance gradually decreases, indicating that it takes a long time to establish an equilibrium state. This leads to the hysteresis of the measured resistance below *T*_cr_.


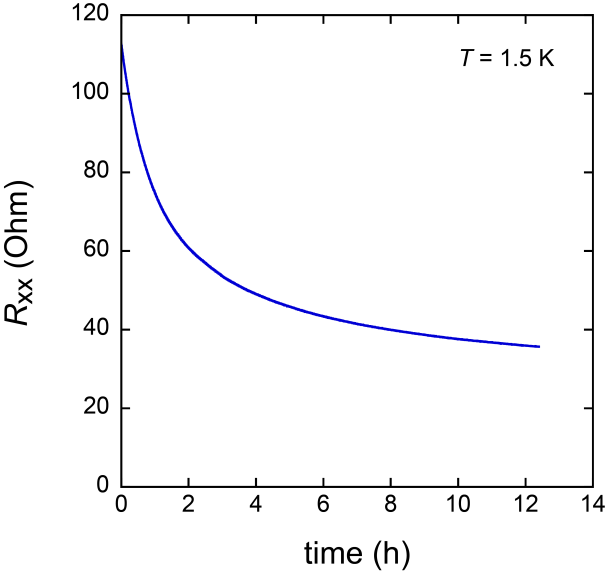


Figure S1. Resistance measured at fixed temperature, 1.6 K, as a function of elapsed time.

2. Hall measurement at low magnetic fields

We measured the Hall effect at magnetic fields *B* in the range from −1 to 1 T.

Figure S2 shows *R*_yx_ as a function of *B*, which shows linear dependence on *B*.


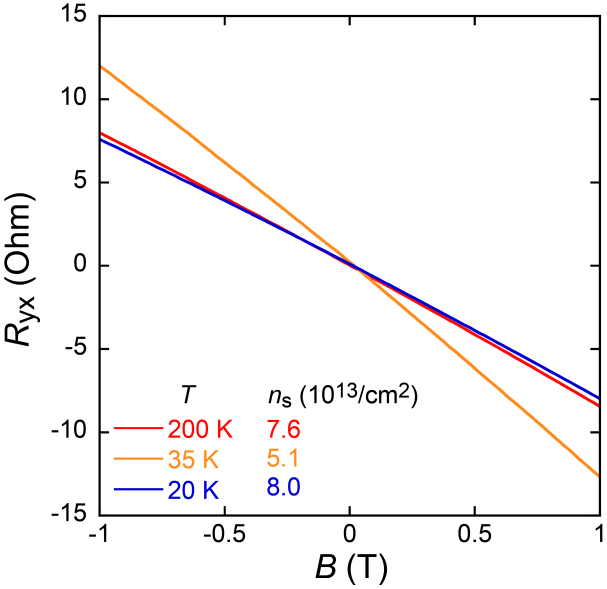


Figure S2. Hall data at low magnetic fields. The values of electron density are indicated.

3. Semi-quantitative analysis for bulk and surface conduction

To separate the conduction in the bulk and the surface, we performed semi-quantitative analysis by subtracting the sheet conductivity of thin sample $\sigma_{s}^{\left( 82 \mathrm{nm} \right)}$ from that of thick sample $\sigma_{s}^{\left( 100 \mathrm{nm} \right)}$. The superscripts show the thickness of the samples. Assuming the sheet conductivity at the surface is identical for two samples, we obtain the sheet conductivity of bulk region of 18 nm from the difference, $\Delta\sigma_{s}^{(18 \mathrm{nm})}\left( \equiv\sigma_{s}^{\left( 100 \mathrm{nm} \right)}-\sigma_{s}^{\left( 82 \mathrm{nm} \right)} \right)$. Furthermore, $\sigma_{s\left( \mathrm{surf} \right)}^{{(2d}_{s} \mathrm{nm})}\equiv\sigma_{s}^{\left( 100 \mathrm{nm} \right)}-\Delta\sigma_{s}^{(18 nm)} \cdot\frac{{100-2d}_{s}}{18} \left( =\sigma_{s}^{\left( 82 \mathrm{nm} \right)}-\Delta\sigma_{s}^{(18 nm)}\cdot\frac{{82-2d}_{s}}{18} \right)$ corresponds to the sheet conductivity at the top and bottom surfaces with total thickness of 2*d*_s_ nm. The value of $d_{s}$ was minimized under the condition that the value of $\sigma_{s\left( \mathrm{surf} \right)}^{(2d_{s} \mathrm{nm})}$ should be positive. Fig. S3 shows temperature dependence of $\Delta\sigma_{s}^{(18 \mathrm{nm})}$ and $\sigma_{s\left( \mathrm{surf} \right)}^{({2d}_{s} \mathrm{nm})}$ for 2*d*_s_ = 20, 25, and 30 nm. Since the value of $\sigma_{s\left( \mathrm{surf} \right)}^{({2d}_{s} \mathrm{nm})}$ is negative for 2*d*_s_ = 20 nm at high temperatures, ${2d}_{s}$ was evaluated to be 25 nm. One possible reason for large surface thickness is that the conductivity in the bulk region $\sim{(\sigma}_{s}-\sigma_{s\left( \mathrm{surf} \right)}^{(2d_{s} \mathrm{nm})})/(t-{2d}_{s})$ is not completely identical for the two samples. However, the different temperature dependence between the bulk and the surface transport is clearly shown in Fig. S3. With decreasing temperature, $\Delta\sigma_{s}^{(18 \mathrm{nm})}$ monotonously decreased above *T*_cr_ and increase below *T*_cr_, while $\sigma_{s\left( \mathrm{surf} \right)}^{(25 \mathrm{nm})}$ slightly decreased from 200 K to 100 K, and gradually increased below 100 K. Thus, the temperature dependence of σ_s_ at the SM transition is same as that of $\Delta\sigma_{s}^{(18 \mathrm{nm})}$.

On the other hand, the behavior of $\sigma_{s\left( \mathrm{surf} \right)}^{({2d}_{s} \mathrm{nm})}(T)$ above *T*_cr_ is similar to that of the field-effect mobility $\mu_{\mathrm{FE}}^{\left( V_{g} >V_{0} \right)}(T)$ shown in Fig. 4(c). This also justifies our premise that $\mu_{\mathrm{FE}}^{\left( V_{g} >V_{0} \right)}(T)$ stands for the mobility of the surface carriers. The increase in $\sigma_{s(\mathrm{surf})}(T)$ below *T*_cr_ can be explained by assuming that $\sigma_{s(\mathrm{surf})}(T)$ contains a portion of bulk transport or that only the top surface has large $\sigma_{s(\mathrm{surf})}$. Based on these assumptions, two mechanisms for the S-M transition are proposed in the main text; one originates from the bulk state, and the other from the top surface state. However, comparing the bulk conductance, $\Delta\sigma_{s}^{(18 \mathrm{nm})}\cdot\frac{t-2d_{s}}{18}$, and surface one, $\sigma_{s\left( \mathrm{surf} \right)}^{({2d}_{s} \mathrm{nm})}$, we notice that the increase in $\sigma_{s}$ below *T*_cr_ is dominantly due to the bulk. In Fig. S3(c), the relation between $\sigma_{s}^{\left( 82 \mathrm{nm} \right)}$ and $\sigma_{s}^{\left( 100 \mathrm{nm} \right)}$ is plotted at each temperature. The relation is fitted with a linear line, $\sigma_{s}^{\left( 82 \mathrm{nm} \right)}=0.802\sigma_{s}^{\left( 100 \mathrm{nm} \right)}$, *i.e.*, the sheet conductance is proportional to the sample thickness at the entire temperature region. This also indicates that the conductivity is mainly determined by the bulk.

(a) (b)


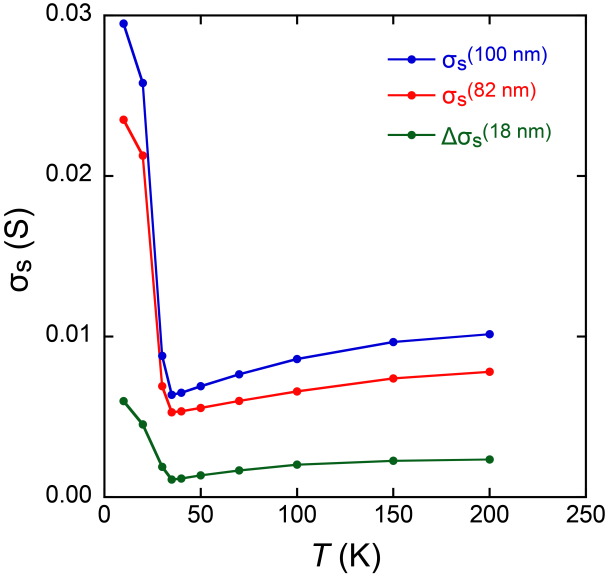

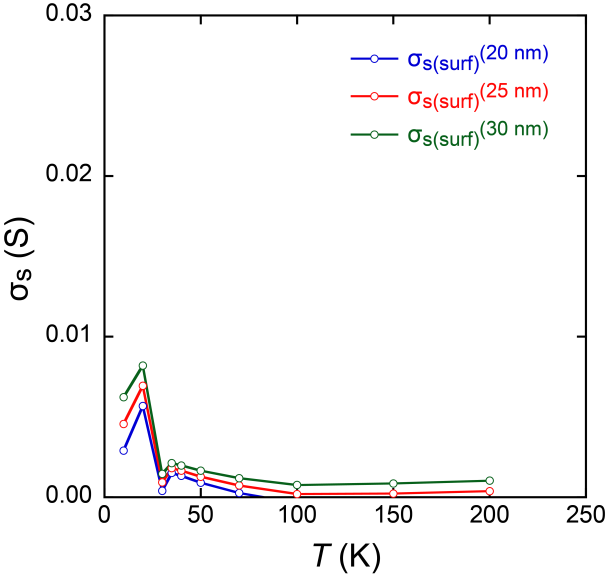


(c)


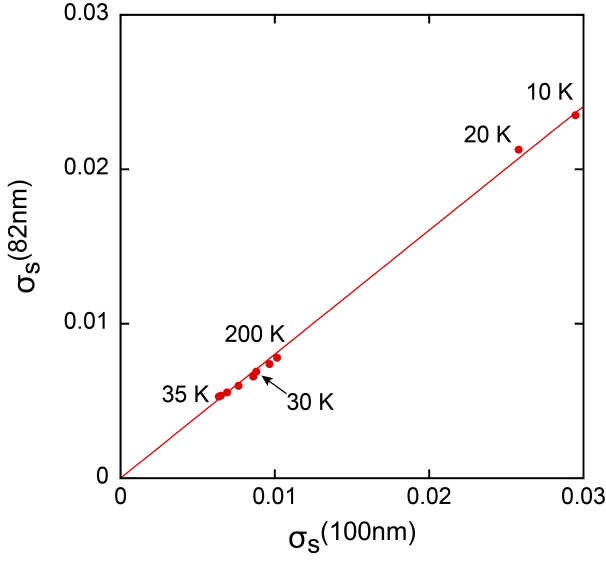


Figure S3. Semi-quantitative analysis for bulk and surface conduction.
